# Designing youth mental health services to improve access: A qualitative study and framework analysis of youths’ perspectives in Singapore

**DOI:** 10.1192/j.eurpsy.2024.269

**Published:** 2024-08-27

**Authors:** J. A. Vaingankar, M. Subramaniam, E. Samari, S. Chang, C. Tang, Y. P. Lee, S. A. Chong, S. Verma

**Affiliations:** ^1^Research Division, Institute of Mental Health; ^2^Saw Swee Hock School of Public Health, NUS; ^3^Department of Psychosis, Institute of Mental Health; ^4^Institute of Mental Health; ^5^Duke-NUS Medical School, Singapore, Singapore

## Abstract

**Introduction:**

Although there is an increasing interest in making mental health services (MHS) accessible to youths, there is limited ground-up involvement of youths while designing MHS in Asian settings.

**Objectives:**

This qualitative study sought to understand what youths considered as important elements of youth centric MHS and how these could be designed to improve access by youths in Singapore.

**Methods:**

We conducted seven focus group discussions, and four semi-structured interviews with 50 multiracial youths aged 15-35 years in Singapore - a high-income Southeast Asian country. Purposive sampling allowed adequate representation of age, gender, and race (mainly Chinese, Malay, and Indian) groups. Participants reflected on the features of an ideal MHS for youths and how these could improve youths’ attitude and access to services. Participants also shared their preferences and additional opinions for culturally tailored and age appropriate MHS. Framework analysis using the ‘Conceptual Framework of Access to Healthcare’ (Levesque et al. Int J Equity Health 2013, 12:18) was used to code transcripts and identify the key themes (Ritchie & Spencer. In Analyzing qualitative data, 1994).

**Results:**

The average age of the participants was 24 years. About one third of the participants had accessed MHS in the past. Three key themes were identified – making facilities ‘approachable’, ‘available and appropriate’ and ‘affordable’. (i) Making facilities approachable related to having non-stigmatizing, non-threatening and welcoming aesthetics, organizational culture, and personnel. The participants also recommended a range of professional services, digital tools, and online features to enhance the approachability of MHS designed for youths. (ii) Flexible operating hours, easy appointment management, accessible location, and easy availability to youths with unique needs (e.g., employed youths) or socio-cultural backgrounds were necessary for making facilities available and accessible to youths. (iii) While sharing challenges of family involvement in the help-seeking process, most of the participants, particularly those in the lower ages, talked about tailoring MHS to the ability of youths to pay for the services. Preferences such as having cheaper services for teenagers and initial contacts, offering more non-medical but trained professionals, and considering shorter in-person counselling sessions, followed by free online options were brought up by the participants.

**Conclusions:**

The study provided insights into multiple aspects of MHS and how these could be designed to cater to the needs of youths in Singapore from their perspective. MHS that incorporate non-stigmatizing, flexible, non-threatening and affordable design approaches could improve help-seeking and early interventions in youths.

**Disclosure of Interest:**

None Declared

